# Extracellular calcium modulates brown adipocyte differentiation and identity

**DOI:** 10.1038/s41598-017-09025-3

**Published:** 2017-08-21

**Authors:** Ines Pramme-Steinwachs, Martin Jastroch, Siegfried Ussar

**Affiliations:** 1 JRG Adipocytes & Metabolism, Institute for Diabetes & Obesity, Helmholtz Center Munich, 85748 Garching, Germany; 2 German Center for Diabetes Research (DZD), 85764 Neuherberg, Germany; 3 Institute for Diabetes & Obesity, Helmholtz Center Munich, 85748 Garching, Germany

## Abstract

Brown adipocytes are important in regulating non-shivering thermogenesis, whole body glucose and lipid homeostasis. Increasing evidence supports an important role of metabolites as well as macro- and micronutrients in brown adipocyte differentiation and function. Calcium is one of the most abundant ions in the body regulating multiple cellular processes. We observed that increasing extracellular calcium concentration during brown adipocyte differentiation blocks lipid accumulation and suppresses induction of major adipogenic transcription factors such as PPARγ and C/EBPα. In contrast, the depletion of calcium in the medium enhances adipogenesis and expression of brown adipocyte selective genes, such as UCP1. Mechanistically, we show that elevated extracellular calcium inhibits C/EBPβ activity through hyperactivation of ERK, a process that is independent of intracellular calcium levels and reversibly halts differentiation. Moreover, increased extracellular calcium solely after the induction phase of differentiation specifically suppresses gene expression of UCP1, PRDM16 and PGC1-α. Notably, depleting extracellular calcium provokes opposite effects. Together, we show that modulating extracellular calcium concentration controls brown adipocyte differentiation and thermogenic gene expression, highlighting the importance of tissue microenvironment on brown adipocyte heterogeneity and function.

## Introduction

Brown adipose tissue (BAT) differs significantly from white adipose tissue (WAT) with respect to function, morphology and developmental origin^1, 2^. Although both tissues are important endocrine organs, the primary function of white adipose tissue is to store energy in form of triglycerides, whereas BAT dissipates energy in form of heat through mitochondrial uncoupled respiration using the uncoupling protein 1 (UCP1)^3–5^. BAT is found in the neck and supraclavicular regions of adult humans^6^, as well as in the interscapular region of infants and rodents^7^. Multilocular lipid droplets and a high density of mitochondria morphologically distinguish brown from white adipocytes and are therefore major characteristics of brown adipocytes. Previous work by Spiegelman^8^ and others^9, 10^ has shown that brown adipocytes originate from distinct, Myf5- positive, precursor populations. However, more recent data indicate that Myf5- positive precursor cells can also give rise to distinct white adipocytes, preferentially within visceral adipose tissue depots, suggesting a complex developmental program distinguishing brown from white adipocytes^11^.

Various extrinsic^12–14^ and endocrine factors^15–17^, such as ambient temperature, bone morphogenetic proteins (BMPs) etc., modulate brown adipocyte differentiation and activity. Interestingly, relatively little is known how the local microenvironment, comprising the extracellular matrix, proximity to blood vessels and local concentrations of nutrients, regulates brown adipocyte differentiation and function. Among the diversity of macro- and micronutrients, calcium is one of the most abundant and important ions in the body. Calcium regulates essential body functions, such as calcification of bones, neuronal transmission, muscle contraction, insulin release and many more^18–21^. To this end, it is not surprising that serum calcium levels are tightly regulated and alterations can have detrimental consequences for human health^22–24^. Local interstitial calcium concentrations, however, are much less studied and could be more variable. There is limited knowledge on the role of extracellular calcium fluctuations in obesity, but reducing excess calcium in the blood by nutritional supplements like calcitonin improves body weight in obese rats^25^. The impact of this reduction on calcium homeostasis and (brown-) adipose tissue function has not been addressed in detail yet.

In contrast, several studies investigated the role of elevating calcium – extracellularly, or via liberation of intracellular calcium stores – on adipogenesis and white adipocyte function, with somewhat contradicting results. Work on human^26^ and murine^27^ white preadipocytes showed that elevation of intracellular calcium levels in early differentiation inhibits the induction of PPARγ and triglyceride accumulation. These effects are most likely mediated through calcium-dependent activation of calcineurin^27^ and calcium/calmodulin-dependent kinase kinases^28^, inhibiting early adipogenic transcription factors. A similar inhibition of white adipocyte differentiation was observed upon elevation of extracellular calcium concentrations, however, without affecting intracellular calcium levels^29^. In contrast, elevation of intracellular calcium concentrations later during adipogenesis promotes lipogenesis and adipocyte marker expression^26^. These effects of extra- and intracellular calcium on adipogenesis seem to be specific to “classical” white adipocytes, as treatment of bone marrow stromal cells (BMSCs) with high extracellular and intracellular calcium accelerates proliferation and differentiation into adipocytes^30^. This differential impact of calcium on the differentiation of BMSCs and white preadipocytes suggests that alterations in the local calcium concentration may have specific impact on individual preadipocyte populations. Given similarities in the transcriptional network mediating white and brown adipogenesis, varying extracellular calcium concentrations may also affect brown adipocyte differentiation and function.

Therefore, we investigated the impact of elevated extracellular calcium throughout different stages of brown adipocyte differentiation. In line with data from white adipocytes, we observed suppressed differentiation of brown adipocytes that have been continuously exposed to high extracellular calcium. Calcium free medium, on the other hand, enhanced brown adipocyte differentiation. In both cases, normalization of extracellular calcium concentrations after the initial induction phase allowed adipocytes to resume differentiation and to accumulate lipids. These effects are independent of increased intracellular calcium levels and calcineurin activity, but at least partially depend on hyperactivation of ERK and regulation of C/EBPβ activity.

## Results

### Extracellular calcium modulates differentiation of brown adipocytes

**Figure 1:**
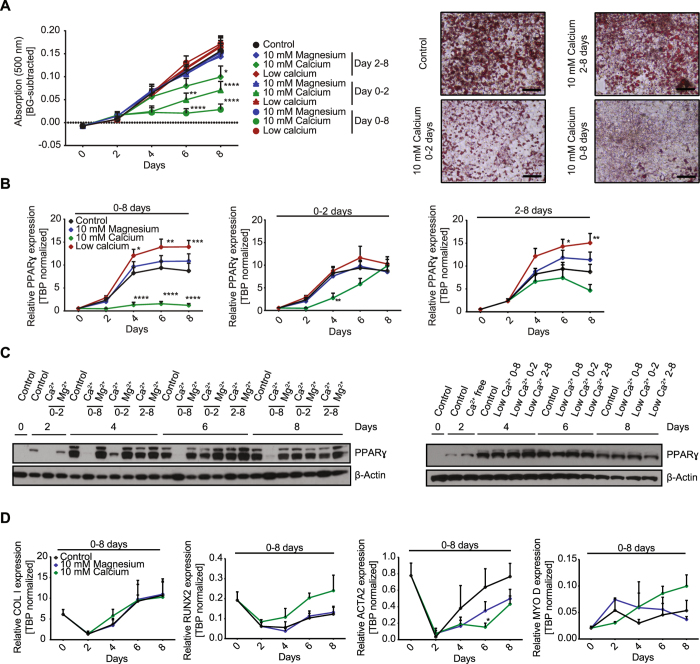
Extracellular calcium modulates differentiation of brown adipocytes. (**A**) Time course of lipid content of *in vitro* differentiated brown adipocytes. Cells were treated with 1.8 mM (control), 10 mM magnesium, 10 mM calcium or calcium free medium for either 8 days (day 0–8), only during induction (day 0–2), or after induction (day 2–8). Shown are background subtracted lipid contents, measured by Oil Red O (500 nm) (n = 6) and representative images of stained cells at day 8 after control or 10 mM calcium treatment. (size bar = 20 µm). (**B**) PPARγ expression normalized on TBP during an eight-day time course under the conditions as above (n = 5). (**C**) Western Blot for PPARγ and β-Actin during the eight- day time course of differentiation with normal 1.8 mM calcium (control), 10 mM calcium (Ca^2+^), 10 mM magnesium (Mg^2+^) and calcium free medium during indicated time points. (**D**) Gene expression of osteogenic markers ColI and Runx2 as well as muscle markers ACTA2 and MyoD normalized on TBP during the eight- day time course of differentiation under indicated conditions (n = 3). For (**A**,**B** and **D**) data are shown as mean ± SEM. Two-way ANOVA with Dunnett’s posthoc test (**A**,**B**) and Tukey’s posthoc test (**D**); *p < 0.05, **p < 0.01, ***p < 0.001, ****p < 0.0001. Full Western Blot images are provided in the Supplemental Information.

To study the impact of extracellular calcium on brown adipocyte differentiation, immortalized murine brown preadipocytes were differentiated for eight days in regular cell culture medium containing 1.8 mM calcium, medium supplemented with 10 mM calcium, or 10 mM magnesium, or medium without calcium (referred to as low calcium due to ~0.3 mM calcium supplemented to all media by the use of 10% FBS). Magnesium was used to control for osmotic and charge effects. Samples were collected every other day during the eight-day time course of differentiation. Treatment of cells with 10 mM calcium throughout differentiation (day 0–8) diminished lipid accumulation as measured by Oil Red O staining (Fig. 1A). In contrast, restricting calcium exposure to days 2–8, following the initial induction phase (days 0–2), only slightly reduced lipid accumulation, with significant differences detected only at day 8 of differentiation. Limiting exposure to high calcium to the initial two days of adipocyte differentiation prevented lipid accumulation during the first four days. Following normalization of extracellular calcium concentrations at day two, brown adipocytes were able to accumulate lipids, albeit the amount remained significantly reduced compared to control cells (Fig. 1A). Exposure to 10 mM magnesium, or the use of calcium free medium did not impair lipid accumulation in brown adipocytes (Figs 1A, S1A). Importantly, none of the treatments impaired cell viability (Figure S1B).

Gene expression analysis of PPARγ (Fig. 1B) and C/EBPα (Figure S1C) confirmed the suppressive effect of extracellular calcium on brown adipocyte differentiation. High extracellular calcium throughout differentiation suppressed PPARγ and C/EBPα mRNA levels, whereas exposure from days 2–8 did not significantly alter expression of these differentiation markers. Exposure to high calcium (10 mM) during the induction phase suppressed PPARγ and C/EBPα expression until day four but normalizing calcium levels restored expression at later time points (Fig. 1B, S1C). Interestingly, differentiation in calcium free medium for either eight days or from days 2–8 significantly increased expression of PPARγ, whereas exposure to high magnesium had no effect compared to cells differentiated in control medium. The decrease in PPARy mRNA expression and its rescue upon calcium normalization were confirmed at the protein level (Fig. 1C, S1D).

Extracellular calcium is known to regulate osteogenic versus adipogenic lineage commitment in BMSCs^31^ and brown adipocytes share common progenitor cells with skeletal muscle. Thus, we investigated the expression of osteogenic markers Collagen I (ColI) and Runt-related transcription factor 2 (Runx2), as well as the myogenic markers α-smooth muscle actin (ACTA2) and myogenic factor 3 (Myf-3, MyoD) in cells differentiated under control conditions, or continuously exposed to either high calcium or high magnesium. Neither osteogenic nor myogenic markers were significantly increased in calcium treated compared to control or magnesium treated cells at any time point during differentiation (Fig. 1D).

### Extracellular calcium regulates C/EBPβ and ERK activity independently of intracellular calcium

**Figure 2:**
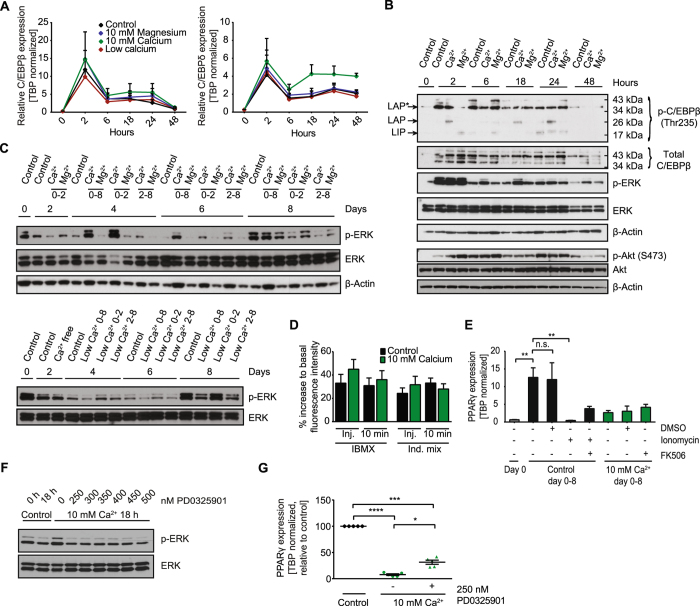
Extracellular calcium regulates MAPKs and C/EBPβ activity independently of intracellular calcium. (**A**) C/EBPδ and C/EBPβ expression normalized to TBP during the first 48 hours of differentiation in 1.8 mM calcium (control), 10 mM magnesium, 10 mM calcium and calcium free medium (n = 6). (**B**) Western Blot for phospho- (Thr235)/total C/EBPβ as well as phospho-/total ERK and phospho- (Ser473)/total AKT during the first 48 hours of differentiation under 1.8 mM calcium (control), 10 mM magnesium (Mg^2+^) and 10 mM calcium (Ca^2+^) conditions with β-Actin as loading control. (**C**) Western Blot for phospho- and total ERK and β-Actin as loading control of the 8 day time course under control (1.8 mM calcium), 10 mM calcium and 10 mM magnesium conditions as well as control and calcium free conditions for the indicated duration. (**D**) Relative increase of intracellular calcium in brown preadipocytes maintained in 1.8 mM calcium (control) or 10 mM calcium directly upon and 10 min after injection of IBMX or induction mix shown as percent increase of fluorescence to basal level. Cells were loaded with the calcium binding fluorophore Fluo-4 (4 µM) and fluorescence was recorded at Ex/Em = 485/520 in orbital averaging (n = 3 with 3–4 replicates each). (**E**) PPARγ expression normalized on TBP in preadipocytes (day 0) and differentiated cells at day 8 treated day 0–8 with 1.8 mM calcium (Control day 0–8) or 10 mM calcium (10 mM Ca^2+^ day 0–8) supplemented with DMSO, ionomycin (2 µM) and/or the calcineurin inhibitor FK506 (1 µM) (n = 3). (**F**) Western Blot for phospho-/total ERK in preadipocytes (0 h) and induced preadipocytes (18 h) with different concentrations of the MEK inhibitor PD0325901. (**G**) PPARγ expression as percent of control in differentiated adipocytes treated with 1.8 mM calcium (control) or 10 mM calcium +/− the MEK inhibitor PD0325901 (250 nM) for 0–8 days (n = 5). For (**A,D,E** and **G**) data are shown as mean ± SEM, *p < 0.05, **p < 0.01, ***p < 0.001, ****p < 0.0001; (**A**) Ordinary two-way ANOVA with Dunnett’s posthoc test, (**D**,**G**) Repeated measures one-way ANOVA with Tukey’s posthoc test and Kruskal-Wallis one-way ANOVA with Dunn’s multiple comparison test (**E**). Full Western Blot images are provided in the Supplemental Information.

The induction of PPARγ expression during early adipogenesis requires promoter binding of C/EBPβ and δ^32^. Alterations of C/EBPβ and δ expression and/or activation have been shown to inhibit adipocyte differentiation in multiple studies^33–35^. Thus, we studied the kinetics of C/EBPβ and δ expression during the first 48 hours of differentiation (Fig. 2A). C/EBPβ and δ expression peaked two hours following the induction of differentiation. However, we did not observe any differences between the treatment groups, except C/EBPδ expression trending to higher levels at later time points in cells treated with high extracellular calcium (Fig. 2A). In contrast, the liver-enriched activator protein (LAP) isoform of C/EBPβ, was hyperphosphorylated throughout the first 24 hours, while the other transcriptionally active C/EBPβ isoform (LAP*) was comparable to controls (Fig. 2B, S2A). Phosphorylation of the inhibitory liver-enriched inhibitor protein (LIP) C/EBPβ isoform was not significantly changed upon calcium treatment. The LAP/LIP ratio is important to regulate adipogenic gene expression^34^, suggesting that the inability to induce PPARy expression upon exposure to high extracellular calcium is most likely due to a shifted pLAP/pLIP ratio.

Phosphorylation of C/EBPβ is primarily mediated through ERK. We observed hyperactivation of ERK upon high extracellular calcium treatment from 6–48 hours, with strongest effects after six hours (Fig. 2B, S2B) and throughout the eight-day time course of differentiation (Fig. 2C, S2C). Interestingly, reducing extracellular calcium decreased ERK phosphorylation throughout the eight-day time course of differentiation, indicating that lower levels of ERK phosphorylation associate with elevated adipocyte differentiation in this experimental setup. In contrast, phosphorylation of Akt, was not significantly altered upon high calcium exposure (Fig. 2B). Regulation of ERK signaling through extracellular calcium via the calcium sensing receptor (CaSR) is very unlikely as its expression is not detectable in our cells, regardless of treatment (Figure S2D). Thus, we hypothesized that raising extracellular calcium could activate the calcium/calmodulin-dependent serine/threonine protein phosphatase calcineurin, which in turn regulates ERK and C/EBPβ phosphorylation and thereby adipocyte differentiation^27, 36, 37^. Cytoplasmic calcium concentrations increased upon the addition of 3-isobutyl-1-methylxanthine (IBMX) or the complete induction mix (Fig. 2D). However, we did not observe differences in cytoplasmic calcium concentrations between control and high extracellular calcium levels nor did the pharmacological inhibition of calcineurin using FK506 rescue adipogenesis (Fig. 2D and 2E). Experiments using calcium free buffer showed that increases in cytosolic calcium result from intracellular calcium store mobilization, being independent of extracellular calcium concentrations (Figure S2E). These data suggest that hyperphosphorylation of ERK and the inhibition of adipogenesis are independent of calcium influx from the extracellular space in our experimental setup.

Based on these results, we investigated if titrating ERK phosphorylation to the levels observed in cells cultured in control medium could rescue brown adipocyte differentiation. We found that 250 nM of the MEK inhibitor PD0325901 reduced ERK phosphorylation to that of cells induced with control medium for 18 hours (Fig. 2F). However, reducing ERK phosphorylation in that way did only partially rescue adipogenesis as indicated by PPARy expression (Fig. 2G).

### Extracellular calcium modulates brown adipocyte identity

**Figure 3:**
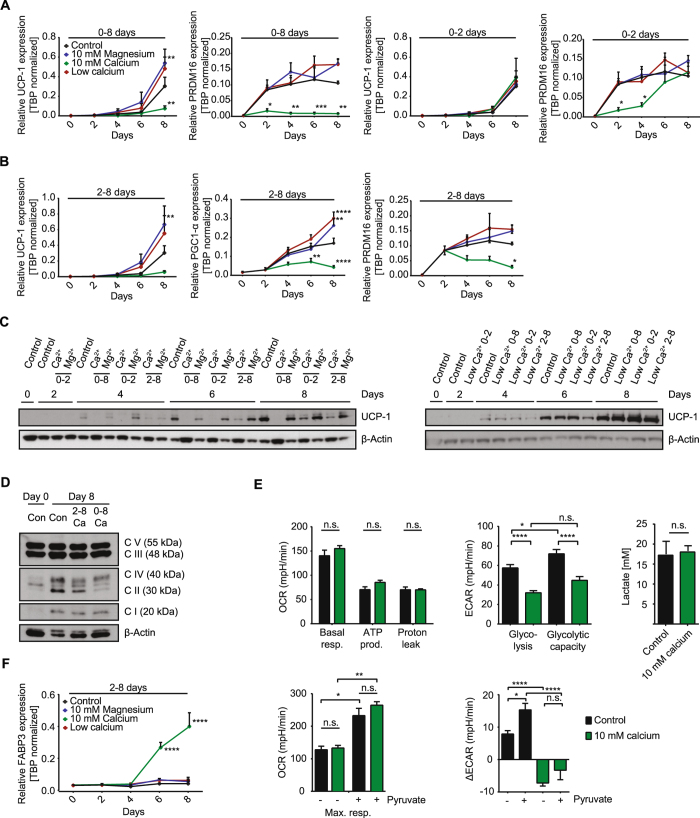
Extracellular calcium modulates brown adipocyte identity and thermogenic markers. (**A**) UCP1 and PRDM16 expression normalized to TBP of *in vitro* differentiated preadipocytes in an eight- day time course under normal 1.8 mM calcium (control), 10 mM calcium,10 mM magnesium and calcium free conditions for days 0–8 and days 0–2 (UCP1, PRDM16 n = 5). (**B**) UCP1, PGC1-α and PRDM16 expression normalized on TBP in these different treatment groups during days 2–8 (UCP1, PRDM16 n = 7, PGC1-α n = 4). (**C**) Western Blot for UCP1 and β-Actin during the eight - day time course of differentiation with normal 1.8 mM calcium (control), 10 mM calcium (Ca^2+^), 10 mM magnesium (Mg^2+^) or calcium free medium during indicated time points. (**D**) OxPhos Western Blot of preadipocytes (Con day 0) and differentiated adipocytes in 1.8 mM calcium (Con) or 10 mM calcium for days 2–8 or days 0–8 with β-Actin as loading control. (**E**) Oxygen consumption rate (OCR) and extracellular acidification rate (ECAR) in differentiated adipocytes at day 8 measured by the Seahorse extracellular flux analyzer. Cells were treated with 1.8 mM calcium (control) or 10 mM calcium for 2–8 days during differentiation. OCR was partitioned into basal respiration, ATP production, proton leak and maximal respiration, also in the presence of pyruvate (5 mM), while ECAR reported glycolytic rate, glycolytic capacity and the difference of glycolytic capacity (ΔECAR) before and after 5 mM pyruvate addition (together with FCCP) (n = 3 for pyruvate n = 2, 3–5 technical replicates each). Lactate concentration of the cell supernatant of 8 day differentiated adipocytes treated with 1.8 mM calcium (control) and 10 mM calcium (2–8 days) (n = 3). (**F**) FABP3 expression normalized to TBP during the time course of differentiation with 1.8 mM calcium (control), 10 mM calcium or 10 mM magnesium for 2–8 days (n = 3). Data are shown as mean ± SEM, *p < 0.05, **p < 0.01, ***p < 0.001, ****p < 0.0001; Ordinary (**A**,**B**) and repeated measures (**F**) two-way ANOVA with Dunnett’s posthoc test. Two-tailed wilcoxon matched-pairs signed rank test (**E**:lactate). (**E**:OCR) Kruskal-Wallis one-way ANOVA with Dunn’s multiple comparison test. (**E**:ECAR) Ordinary one-way ANOVA with Tukey’s posthoc test. Full Western Blot images are provided in the Supplemental Information.

As shown for white adipocytes, brown adipocyte differentiation is inhibited by prolonged elevation of extracellular calcium concentrations. Thus, similarly to general markers of adipogenesis, induction of uncoupling protein 1 (UCP1), PR domain containing 16 (PRDM 16) and PPARγ coactivator 1-alpha (PGC1-α) was inhibited during differentiation upon continued exposure to high extracellular calcium, but this recovered upon removal after day 2 (Fig. 3A, S3A). Reducing calcium in the medium or adding magnesium during differentiation showed trends to elevated expression of UCP1, PRDM16 and PGC1-α, in line with PPARγ expression (Fig. 3A, S3A). Conversely, elevation of extracellular calcium following adipogenic induction did not impair lipid accumulation or adipogenic marker expression, but completely inhibited gene expression of UCP1 and other brown adipocyte marker genes such as PGC1-α and PRDM16 (Fig. 3B). Importantly, the increased UCP1 mRNA levels under low calcium conditions and decreased expression under high calcium were even more pronounced on protein level, whereas full recovery of UCP1 after normalization of calcium levels was not observed at the protein level (Fig. 3C, S3C). Among many other functions, PRDM16 and PGC1-α regulate mitochondrial biogenesis. To this end, we analyzed mitochondrial abundance and function in brown adipocytes exposed to high extracellular calcium from days 2–8 and to controls. Mitochondrial transcription factor A (TFAM) was slightly increased, suggesting some increase in mitochondrial content during differentiation, but without any differences between the treatment groups (Figure S3B). Western Blots for subunits of all five complexes of the mitochondrial respiratory chain (complex I–V) revealed a slight reduction in the expression of some subunits of high calcium treated groups compared to controls at day 8 of differentiation (Fig. 3D, S3D). However, we could not observe significant differences in mitochondrial respiration as assessed by plate-based respirometry using a Seahorse extracellular flux analyzer (Fig. 3E and S3E). ATP synthesis, proton leak and maximal respiration, also upon additional substrate supply by pyruvate addition, were not altered in calcium exposed cells (Fig. 3E and S3E). However, cells differentiated in medium containing high extracellular calcium from days 2–8 exhibited lower extracellular acidification rates, albeit equal supernatant lactate concentrations among groups (Fig. 3E). FABP3 expression correlates with increased long-chain fatty acid uptake and β-oxidation in BAT of UCP1 knockout mice^38–41^. In our cell model, FABP3 expression was strongly induced during differentiation in cells exposed to high extracellular calcium from days 2–8 and when cells were continuously exposed to calcium (Fig. 3F and S3F). Similar to cells continuously exposed to calcium, we also observed a trend to increased MyoD at expression at day 8 in cells treated form 2–8 with 10 mM calcium (Figure 1D,S3G).

## Discussion

Calcium plays an important role in multiple cellular processes. Here we show that modulation of extracellular calcium concentration and duration of exposure regulates brown adipocyte differentiation and key components of the brown adipocyte cell lineage. In line with previous data on white adipocytes^29, 34^, we confirm that continuous exposure to high extracellular calcium inhibits lipid accumulation and the induction of PPARγ and C/EBPα, both being drivers of adipogenic fate. However, exposure to high calcium during the induction phase does not alter lineage commitment in favor towards osteoblasts or myocytes. Conversely, high extracellular calcium reversibly pauses adipogenesis right after lineage commitment, as removal of the calcium block after initiation restores lipid accumulation and adipogenic marker expression. Pausing adipocyte differentiation appears to be mediated through inhibition of C/EBPβ and δ activity, which are key transcription factors initiating adipocyte differentiation through the induction of PPARγ and C/EBPα^42^. We show that elevated extracellular calcium alters the ratio of phosphorylation of the activating C/EBPβ isoforms LAP and LAP* and the inhibitory LIP. C/EBPβ activity is primarily activated by ERK in response to insulin^35, 43, 44^. However, prolonged ERK signaling can also inhibit adipocyte differentiation^45^. Thus, the continuous hyperactivation of ERK, observed in response to elevated extracellular calcium, may explain both the inhibition and the restoration of adipocyte differentiation. Indeed, pharmacologically reducing ERK activity to control cell levels partially restored brown adipocyte differentiation, albeit the differentiation capacity was much lower than in control cells. The diminished ability of this approach to restore adipocyte differentiation could be in part explained by the fact that pharmacological MEK inhibition reduces overall phospho-ERK levels and does not account for spatial and temporal differences in phospho-ERK within different signaling complexes. At this stage, we cannot exclude that other signaling pathways also contribute to the regulation of adipogenesis by extracellular calcium. However, in addition to the inhibitory role of elevated extracellular calcium, we find that reducing calcium in the medium (i) enhances adipocyte differentiation and (ii) reduces ERK phosphorylation during the time course of differentiation.

The upstream signals mediating increased ERK phosphorylation remain to be determined. In the context of calcium, it is feasible to speculate that the calcium sensitive phosphatase calcineurin, which is known to modulate ERK activity^27, 36^, mediates these effects. However, we do not detect a rise in intracellular calcium concentrations in response to elevated extracellular calcium that would be necessary to activate calcineurin. Moreover, inhibition of calcineurin does not restore brown adipocyte differentiation. These findings are in line with previous data from Jensen and colleagues showing that high extracellular calcium inhibits adipogenesis in 3T3-L1 cells without increasing intracellular levels^29^, suggesting that extracellular calcium acts outside the cell to modulate the activity of transmembrane proteins. The G-protein coupled calcium sensing receptor (CaSR) for example was shown to be expressed in adipocytes and to activate intracellular signaling cascades especially ERK upon calcium binding^46^. However, we did not detect expression of CaSR in neither isolated brown adipocytes nor in our cell model. Further, we did not observe an increase in intracellular calcium associated with CaSR activation. Thus, while an involvement of this receptor seems to be very unlikely, it is presumable that calcium promotes receptor binding to ligands, such as integrins or cadherins, which in turn would alter intracellular signaling. Overall our data suggest a very general role of extracellular calcium during the early phase of adipocyte differentiation, which appears to be conserved between white and brown adipocytes. In future studies, it may also be addressed why bone marrow derived adipocytes seem to respond differently.

Nevertheless, we find that extracellular calcium has a very specific effect on brown adipocytes at later differentiation stages. Exposure to high extracellular calcium after the induction phase inhibits the expression of brown adipocyte specific genes such as UCP1, PRDM16 and PGC1-α with little to no impact on the expression of general adipogenic markers and lipid accumulation. As we did not observe differences in mitochondrial respiration between the treatment groups, UCP1 activity is either too low or requires activation as suggested previously^47^ in our cellular model. Alternatively, FABP3 was shown to accelerate β-oxidation and free fatty acid uptake independent of UCP1 and to mediate uncoupled respiration^38, 41^. Thus, it is tempting to speculate that the observed increase in expression of FABP3 in cells treated for eight days or from days 2–8 with 10 mM calcium, could provide an alternative explanation for the lack of differences in mitochondrial respiration, albeit changes in UCP1 expression. The induction of FABP3, which is predominantly expressed in cardiomyocytes and myocytes^39^, could also suggest a role of calcium in determining lineage commitment between muscle and brown adipocytes. However, we did not observe substantial expression of muscle specific genes, albeit prolonged exposure could be required to fully dissect this phenomenon.

In conclusion, we provide evidence that manipulation of extracellular calcium as an important constituent of the microenvironment can have profound effects on the kinetics and nature of *de novo* adipogenesis. To date, different biotechnological platforms aim to establish protocols to culture and expand patient-derived human brown adipose tissue *ex vivo* for allograft transplantation. To this end, our data show that omitting calcium in the cell culture medium may substantially improve brown adipocyte differentiation and expression of the thermogenic program. Conversely, our study did not address whether alterations in local calcium concentrations modulate the function of brown adipose tissue *in vivo*, especially in humans. However, it is tempting to speculate that altered local calcium concentrations could contribute to the lower brown fat mass and activity of obese human subjects^22^.

## Methods

### Cell isolation and culture

Murine brown preadipocytes were isolated according to^48^ from adult eight week old C57Bl/6 mice. In detail, murine brown adipose tissue (BAT) was dissected from the mouse interscapular region, cut into ~1mm pieces and digested for 30–45 minutes at 37 °C in DMEM containing 1 mg/ml collagenase type IV (Gibco) and 10 mg/ml BSA (Albumin fraction V, Roth). Digestion was stopped by washing the cells with PBS (Gibco) containing 10 mg/ml BSA. Mature adipocytes were removed and preadipocytes cultured in Dulbecco’s Modified Eagle Medium (DMEM + GlutaMAX + high glucose, Gibco). Cells were immortalized using an ecotropic SV40 large T retrovirus. Animal experiments were conducted in accordance with the German animal welfare law. The sacrifice of the mouse was performed with permission and in accordance with all relevant guidelines and regulations from the Sachgebiet 54-Tierschutz of the district government of Upper Bavaria (Bavaria, Germany).

For all cell culture experiments normal growth medium containing penicillin (100 Units/ml) and streptomycin (100 µg/ml) (Pen Strep, Gibco) as well as 10% fetal bovine serum (FBS, Gibco) were used, supplemented with calcium chloride (high calcium) or magnesium chloride (high magnesium) to reach a final concentration of 10 mM respectively. Low calcium experiments were conducted using DMEM, high glucose, no glutamine, no calcium (Gibco 21068) supplemented with 1 mM sodium pyruvate (Gibco), 1x GlutaMAX^TM^-1 (Gibco) [equimolar with 2 mM L-alanyl-L-glutamine], Pen Strep and 10% FBS (containing 3.5 mM calcium). Immortalized brown preadipocytes were grown up to 100% confluency (day 0) and differentiation was induced with 0.5 mM 3-Isobuthyl-1-methylxanthin (IBMX, Sigma), 125 µM Indomethacin (Santa Cruz), 5 µM Dexamethasone (Sigma), 100 nM Insulin (Sigma) and 1 nM Triiodothyronine (T3, Calbiochem) in the appropriate medium. Medium (supplemented with insulin and T3) was changed every other day until day 8.

For inhibitor experiments the compounds were kept as 1 mM (FK506, Ionommycin) and 10 mM (PD325901) stock solutions in DMSO at −20 °C and supplemented to the medium in the appropriate dilution: 2 µM Ionomycin calcium salt (Fisher Scientific), 1 µM calcineurin inhibitor FK506 (*Tacrolismus* - Abcam), 250 nM MEK inhibitor PD0325901 (Sigma).

### Oil Red O stain

Differentiated cells were fixed in 10% formalin (Roth), washed in water and dehydrated in 60% isopropanol. Then, filtered 60% (v/v) Oil Red O working solution in water (stock: 0.35% (w/v) Oil Red O (Alfa Aesar) in 100% isopropanol (Sigma)) was added, incubated for 10 minutes at room temperature, washed with water and dried. The stain was solved with 100% isopropanol and the absorption at 500 nm was measured with a PHERAstar FS detection system (BMG Biotech) referring the relative lipid content of the cells.

### qPCR

RNA extraction (RNeasy Mini Kit, Qiagen) and cDNA synthesis (0.5–1 µg total RNA, High Capacity cDNA Reverse Transcription Kit, Applied Biosystems) were conducted according to the manufacturers’ instructions. qPCR was performed in a C1000 Touch Thermal Cycler (Bio Rad), using 300 nM forward and reverse primers and iTaq Universal SYBR Green supermix (BioRad). Target gene expression was normalized on the expression of the housekeeping gene TATA box binding protein (TBP)^49^. The primer sequences are shown in Supplemental Table 1. Calculations for relative expression (RE) of genes of interest (GOI) were performed as follows: 2^CT(TBP)-CT(GOI)^.

### Western Blot

Cells were lysed in 25 mM Tris pH 7.4, 150 mM NaCl, 1% NP-40, 1 mM EDTA, 5% glycerol, 0.1% SDS containing protease inhibitor and phosphatase inhibitors (Sigma). 15–20 µg of protein (15–30 µg for UCP1 detection) were loaded on 4–12% precast SDS gels (Invitrogen) or on 10% acrylamide-SDS-gels and blotted on 0.45 µm PVDF membranes, which were blocked in 5% BSA in TBS with 0.1% Tween 20 (TBS-T) one hour at room temperature (2 hours for UCP1). Membranes were incubated with primary antibodies (list in Supplemental Table 2) over night at 4 °C, washed in TBS-T and incubated with the appropriate HRP coupled secondary antibodies one hour at room temperature. Membranes were developed with chemiluminescent HRP substrate (Immobilon Western, Millipore) using films (Hyperfilm ECL, GE Life Sciences; CL-XPosure Film, Thermo Scientific). Quantification of Western Blots was performed using ImageJ software.

### Calcium measurement with Fluo-4

Cells were grown to 100% confluency (day 0) in 96 well glass-bottom plates (Eppendorf) to measure fluorescence with a PHERAstar FS (BMG Biotech) using bottom optic of the optic module FI 485 520 (Ex/Em), adjusting gain to 915 with 20 flashes per well in an orbital averaging mode circling in 3 mm diameter. All further steps were performed at 37 °C with pre-warmed dilutions in the control buffer, 1x SBS (5 mM KCl, 140 mM NaCl, 8 mM glucose, 10 mM HEPES, 0.8 mM MgCl_2_, 1.8 mM CaCl_2_, pH 7,4). For high magnesium (10 mM), high calcium (10 mM) and calcium free (0 mM) experiments specific 1x SBS buffers containing the appropriate magnesium and calcium concentrations were prepared. Cells were washed in SBS buffer containing 1 mM probenecid (Santa Cruz) and incubated with 4 µM Fluo-4 AM (Cat. #F14201, Thermo Scientific), 0.02% Pluronic® F-127 (Biotium) and 1 mM probenecid (Santa Cruz) for one hour at 37°C and washed again. After 30 min, basal fluorescence was detected in intervals of several seconds. IBMX or whole induction mix diluted in calcium free 1x SBS (final concentrations under *Cell isolation and culture*) were injected with a speed of 190 µl/s. After orbital mixing (100 rpm) the fluorescence emission at 520 nm was detected. Values are shown as fold increase in fluorescence intensity (%) upon injection.

### Cellular oxygen consumption and extracellular acidification rates

Cells were seeded in Seahorse 96-well plates and differentiated for eight days with 1.8 mM calcium (control), high magnesium (10 mM), high calcium (10 mM) or calcium free conditions for 2–8 days. The oxygen consumption rate (OCR) and the extracellular acidification rate (ECAR) were measured with a XF96 Extracellular Flux analyzer (Seahorse Bioscience, Agilent technologies). Cells were equilibrated at 37 °C in XF Assay Medium Modified DMEM (Seahorse Bioscience) supplemented with 25 mM glucose one hour prior measurement. All compounds were diluted as ten-fold concentrations in assay medium and loaded in the equilibrated cartridge ports as follows: A) 20 µg/ml oligomycin (Merck), B) 10 µM FCCP (R&D systems) +/− 50 mM sodium pyruvate (Gibco), C) 25 µM rotenone and antimycin A (Sigma), D) 1 M 2-deoxy-D-glucose (Alfa Aesar). Each cycle comprised two minutes each of mixing, waiting and measuring. Non-mitochondrial respiration (OCR) and non-glycolytic acidification (ECAR) were subtracted from other values to determine mitochondrial oxygen uptake. For the analysis, the mean values of either the last two cycles prior injection (basal respiration, acidification/glycolysis) or the first two after injection (sum of ATP production and proton leak or maximal respiration (OCR) and maximal acidification/glycolytic capacity (ECAR)) were averaged and calculated.

### Lactate Assay

Cells were differentiated in 1.8 mM calcium (control) or for day 2–8 in high calcium (10 mM) medium. The cell supernatant at day 8 was diluted and lactate was measured with the Lactate Colorimetric/Fluorometric Assay Kit (Biovision).

### Statistical analysis

Data are presented as means ± SEM. All statistical analyses were performed with GraphPad Prism. Samples were tested for normal distribution with either D’Agostino-Pearson omnibus normality test, Shapiro-Wilk normality test or Kolmogorov-Smirnov test with Dallal-Wikinson Lillie for P value. Parametric or nonparametric tests were performed accordingly as indicated in the figure legends. Two-tailed Student’s t test, one-way and two-way ANOVA for multiple comparisons were used with α-level at 0.05 to determine P values.

### Data availability

The datasets generated during and/or analyzed during the current study are available from the corresponding author on reasonable request.

## Electronic supplementary material

Supplementary Information
