# Supplementary material for: Extracellular calcium modulates brown adipocyte differentiation and identity
Source: Sci Rep. 2017 Aug 21;7:8888. doi: 10.1038/s41598-017-09025-3 (PMC5567186; doi:10.1038/s41598-017-09025-3)

# Supplemental information to:

# Extracellular calcium modulates brown adipocyte differentiation and identity

Ines Pramme-Steinwachs^1,2^, Martin Jastroch^2,3^, Siegfried Ussar^1,2^

^1^JRG Adipocytes & Metabolism, Institute for Diabetes & Obesity, Helmholtz Center Munich, 85748 Garching, Germany, ^2^German Center for Diabetes Research (DZD), 85764 Neuherberg, Germany, ^3^Institute for Diabetes & Obesity, Helmholtz Center Munich, 85748 Garching, Germany

**Supplemental Figure 1
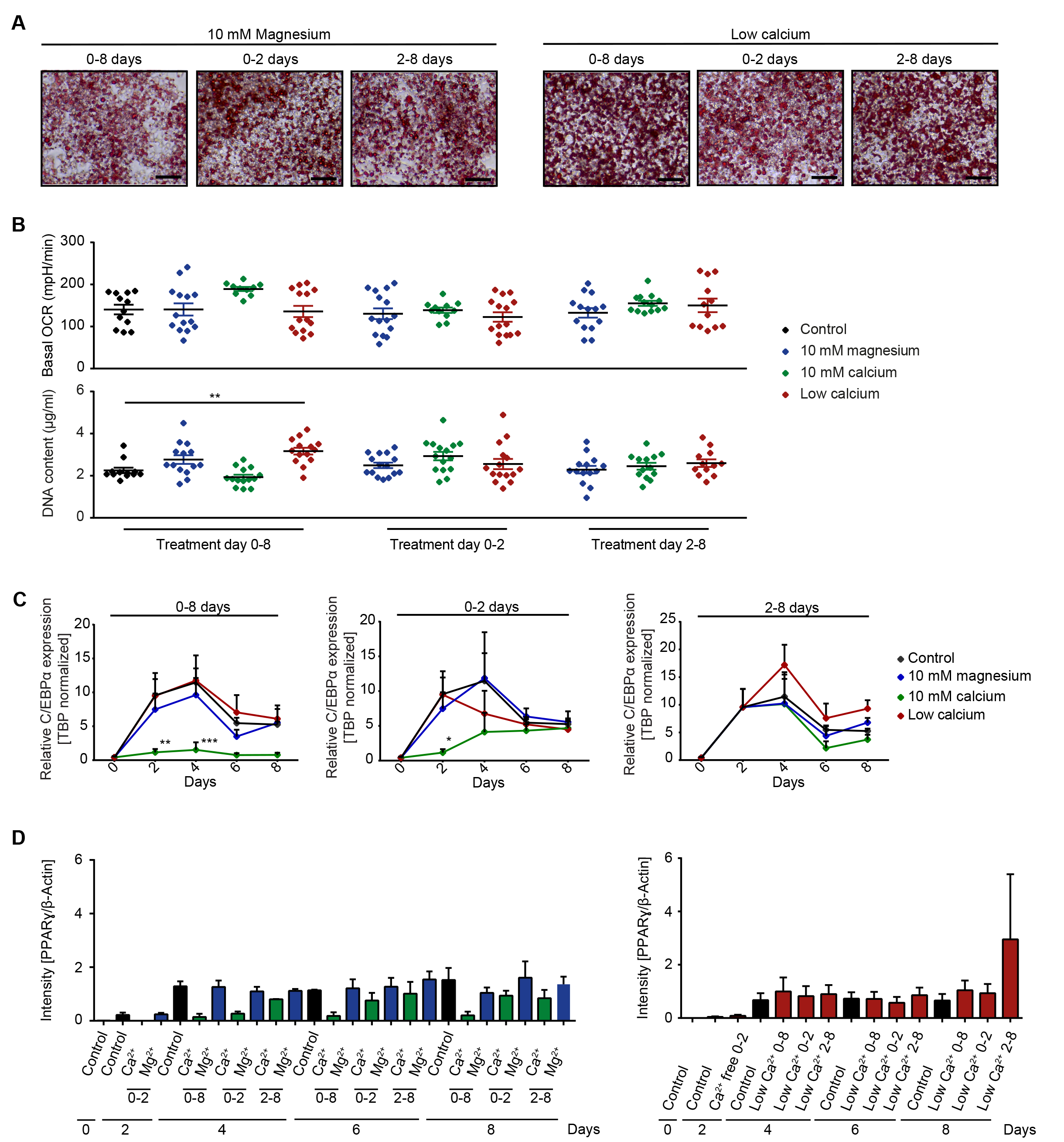
**

**Supplemental Figure 1: (A)** Representative pictures of Oil Red O stained adipocytes at day 8 of differentiation under 10 mM magnesium or low calcium conditions for the indicated duration, size bar = 20 µm. **(B)** Basal oxygen consumption (OCR) and DNA content in cells at day 8 treated under indicated conditions (n=2-3, 3-5 technical replicates each). **(C)** C/EBPα expression normalized on TBP of *in vitro* differentiated preadipocytes during an eight day time course under normal 1.8 mM calcium (control), 10 mM calcium (Ca^2+^), 10 mM magnesium (Mg^2+^) and low calcium conditions during days 2-8 (n=3). **(D)** Quantification of PPARγ protein content normalized to β-actin (n=3). Data are shown as mean ± SEM, * p<0.05, ** p<0.01, *** p<0.001, **** p<0.0001; Ordinary One-way with Holm-Sidak’s multiple comparison test (B top), Kruskal-Wallis one-way ANOVA with Dunn’s multiple comparison test (B bottom) and two-Way ANOVA with Dunnett’s posthoc test (C).

**Supplemental Figure 2**


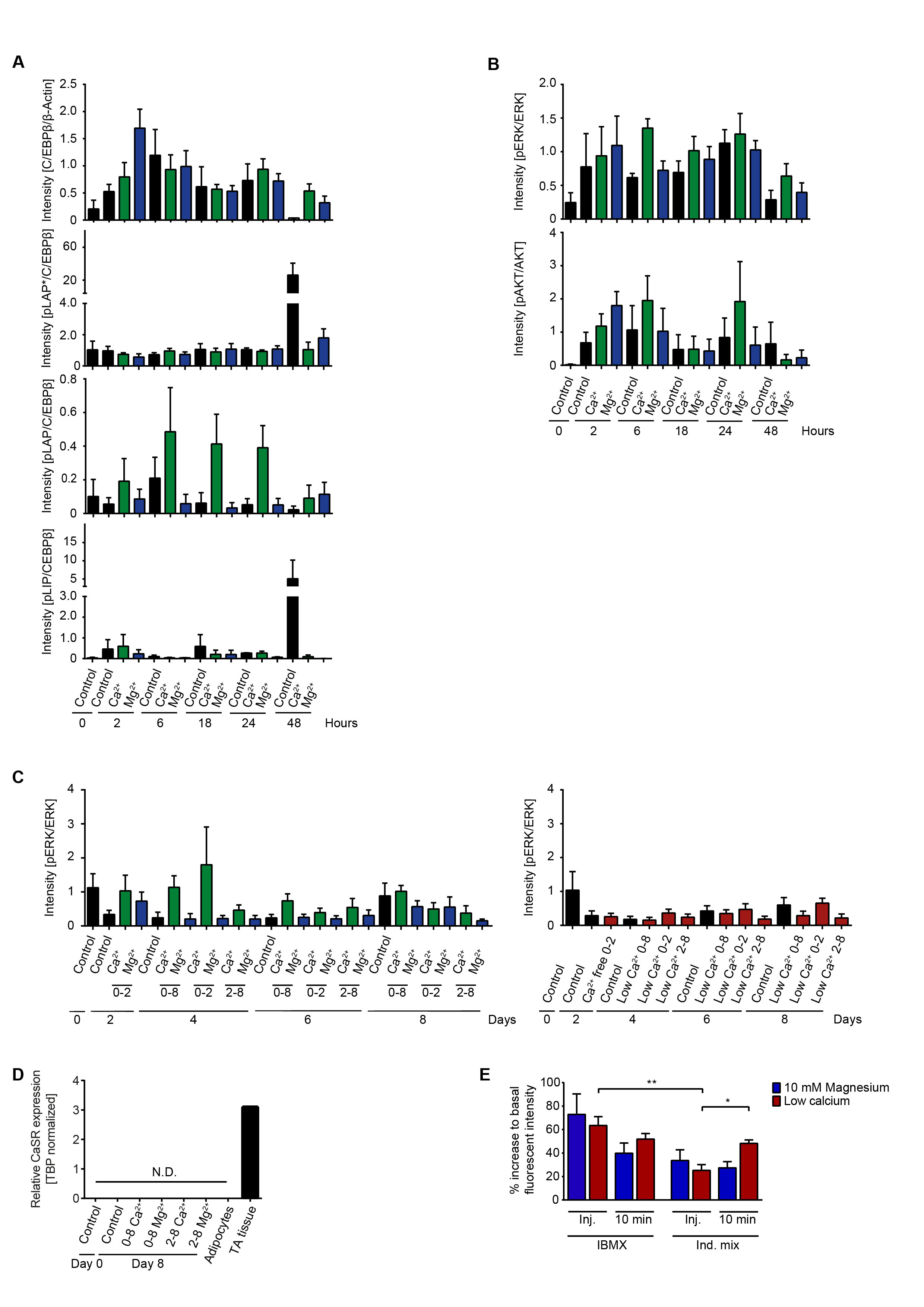


**Supplemental Figure 2:** Quantification of indicated protein signals in western blot normalized on β-actin or total protein during 48 hour time course: (A) Total C/EBPβ to β-Actin (n=4), pLAP*, pLAP and pLIP C/EBPβ to total C/EBPβ (n=3-4); (B) pERK to toal ERK (n=4), pAKT (S473) to total AKT (n=2-3). (C) Quantification of pERK signal normalized to total ERK signal during 8 day time course (n=4). (D) CaSR expression normalized to TBP in preadipocytes (day 0) and in vitro differentiated cells at day 8 under 1.8 mM calcium (control), 10 mM calcium (Ca2+) or 10 mM magnesium (Mg2+) for day 0-8 or 2-8 (n=3) as well as in primary mature adipocytes and murine muscle (tibia anterior –TA) (pooled samples n=1). (E) Relative increase of intracellular calcium in brown preadipocytes kept in 10 mM magnesium or calcium free medium directly upon and 10 min after injection of IBMX or induction mix shown as percent increase to basal level. Cells were loaded with the calcium binding fluorophore Fluo-4 (4 µM) and change in fluorescence was recorded at Ex/Em = 485/520 in orbital averaging (n=3 with 3-4 replicates each). Data are shown as mean ± SEM, * p<0.05, ** p<0.01, *** p<0.001, **** p<0.0001; (E) Repeated measures one-way ANOVA with Tukey’s posthoc test.

**Supplemental Figure 3**

**
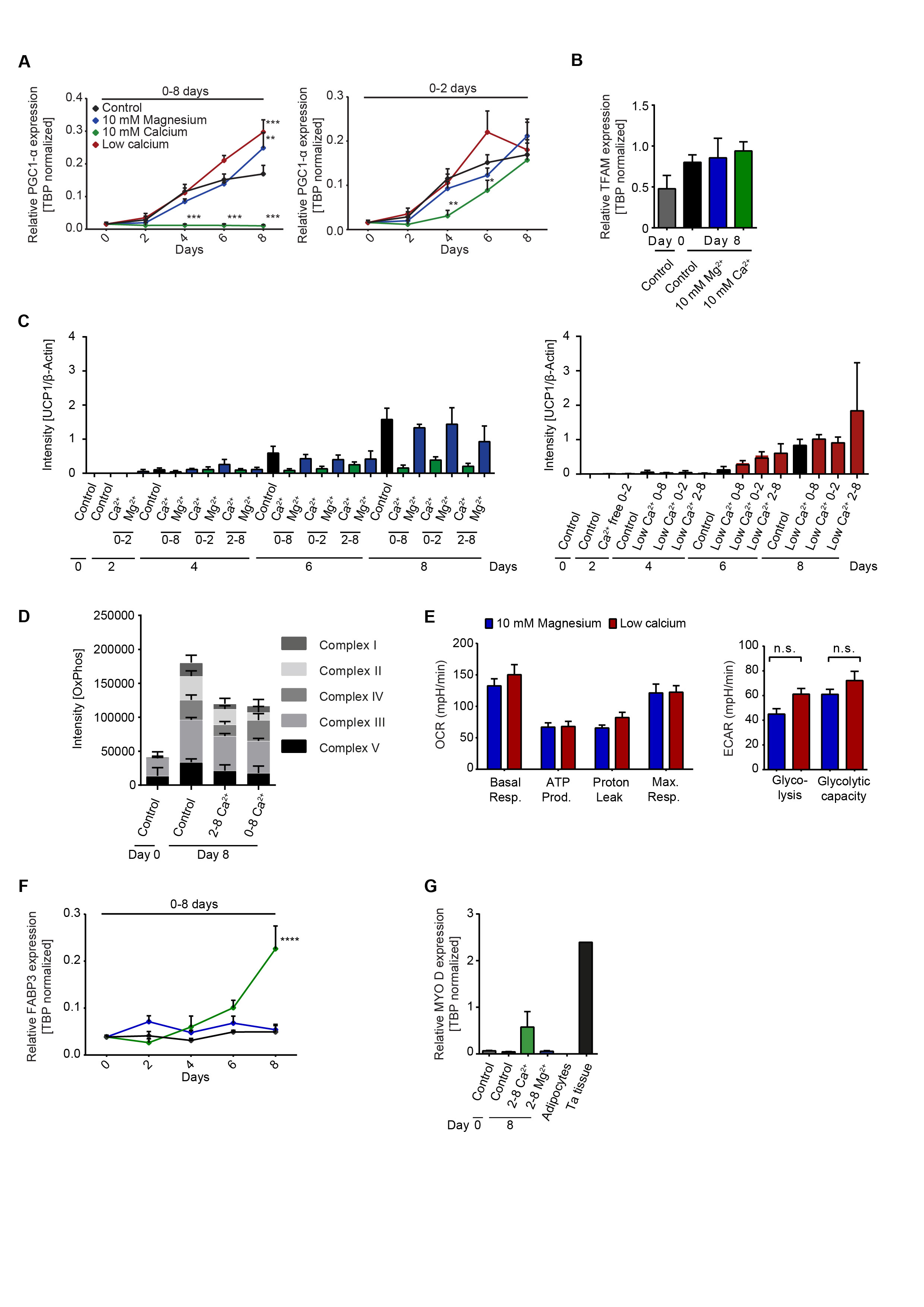
**

**Supplemental Figure 3: (A)** PGC1-α expression normalized to TBP during the eight day time course with the various treatment groups for day 0-8 and day 0-2 (n=4). **(B)** TFAM expression normalized on TBP in preadipocytes (day 0) and in vitro differentiated preadipocytes under normal 1.8 mM calcium (control), 10 mM calcium (Ca^2+^),10 mM magnesium (Mg^2+^) and calcium free conditions during day 2-8 (n=4). **(C)** Quantification of UCP1 protein content normalized to β-actin (n=3). **(D)** Quantification of mitochondrial complexes I-V protein content (n=3; complex, I n=1). **(E)** Oxygen consumption rate (OCR) and extracellular acidification rate (ECAR) measured by the Seahorse extracellular flux analyzer in differentiated adipocytes at day 8 and shown as values with subtracted non-mitochondrial respiration (OCR) and non-glycolytic acidification (ECAR). Cells were treated with 10 mM magnesium or calcium free medium for 2-8 days. During measurement basal respiration, ATP production, proton leak and maximal respiration in dependence of pyruvate addition (5 mM) as well as glycolytic rate and glycolytic capacity were detected (n=3 and n=2 for pyruvate, 6-7 technical replicates each). **(F)** FABP3 expression normalized with TBP during the time course of differentiation with 1.8 mM calcium (control), 10 mM calcium or 10 mM magnesium for 0-8 days (n=3). **(G)** MyoD expression normalized to TBP in preadipocytes (Control d0), in *in vitro* differentiated cells (day 8) under 1.8 mM calcium (control) or 10 mM calcium/magnesium for day 2-8 (n=3) and in isolated mature brown adipocytes as well as in murine muscle (tibia anterior –TA) (pooled samples n=1). Data are shown as mean ± SEM, * p<0.05, ** p<0.01, *** p<0.001, **** p<0.0001; (A, F) Two-Way ANOVA with Dunnett’s posthoc test. Ordinary one-way ANOVA with Tukey’s posthoc test (E) and Friedman one-way ANOVA with Dunn’s multiple comparison test (B).

**Supplemental Table 1:** qPCR primer sequences 5’-3’.

| **Target gene** | **Forward sequence** | **Reverse sequence** |
| --- | --- | --- |
| **CaSR** | **GCTTTTCACCAACGGGTCCT** | **CCTGCTCCCCCATGTTGTT** |
| C/EBPα | AGGTGCTGGAGTTGACCAGT | CAGCCTAGAGATCCAGCGAC |
| C/EBPβ | CCAAGAAGACGGTGGACAA | CAAGTTCCGCAGGGTGCT |
| C/EBPδ | ATCGACTTCAGCGCCTACA | GCTTTGTGGTTGCTGTTGAA |
| Col I | GAAGCCGAGGTCCCAGTG | CACCCCTCTCTCCTGGAAG |
| FABP3 | AGAGTTCGACGAGGTGACAG | TGCACATGGATGAGTTTGCC |
| MYO D | TACAGTGGCGACTCAGATGC | GTGTCGTAGCCATTCTGCC |
| PGC1-α | AGCCGTGACCACTGACAACGAG | GCTGCATGGTTCTGAGTGCTAAG |
| PPARγ | CCCTGGCAAAGCATTTGTAT | GAAACTGGCACCCTTGAAAA |
| PRDM16 | CCGCTGTGATGAGTGTGATG | GGACGATCATGTGTTGCTCC |
| RUNX2 | CTCTGGCCTTCCTCTCTCAG | TGAAATGCTTGGGAACTGCC |
| ACTA2 | CTGTCAGGAACCCTGAGACGC | GGATGGGAAAACAGCCCTGG |
| TBP | ACCCTTCACCAATGACTCCTATG | TGACTGCAGCAAATCGCTTGG |
| TFAM | CAGGAGGCAAAGGATGATTC | CCAAGACTTCATTTCATTGTCG |
| UCP-1 | CTGCCAGGACAGTACCCAAG | TCAGCTGTTCAAAGCACACA |

**Supplemental Table 2:** Primary antibodies for western blot analysis.

| **Protein** | **Company** | **Catalog number** |
| --- | --- | --- |
| Akt | Cell Signaling | #4685 |
| Anti-mouse IgG, HRP coupled | Santa Cruz | sc-2005 |
| Anti-rabbit IgG, HRP coupled | Cell Signaling | #7074 |
| β-Actin, HRP coupled | Santa Cruz | sc-47778 |
| C/EBPβ | Cell Signaling | #3087 |
| p44/42 MAPK (Erk1/2) | Cell Signaling | #4695 |
| PPARγ | Cell Signaling | #2435 |
| p-Akt (S473) | Cell Signaling | #9271 |
| p-C/EBPβ (Thr235) | Cell Signaling | #3084 |
| p-p44/42 MAPK (Erk1/2) | Cell Signaling | #4377 |
| OxPhos Complex Kit | Novex | 458099 |
| UCP-1 antibody | custom* | Rabbit anti-hamster UCP1 |

* A detailed characterization of the antibody can be found in: [Functional characterization of UCP1 in mammalian HEK293 cells excludes mitochondrial uncoupling artefacts and reveals no contribution to basal proton leak.](https://www.ncbi.nlm.nih.gov/pubmed/22676960), Jastroch M, Hirschberg V, Klingenspor M., Biochim Biophys Acta. 2012 Sep;1817(9):1660-70

**Uncropped western blots:**


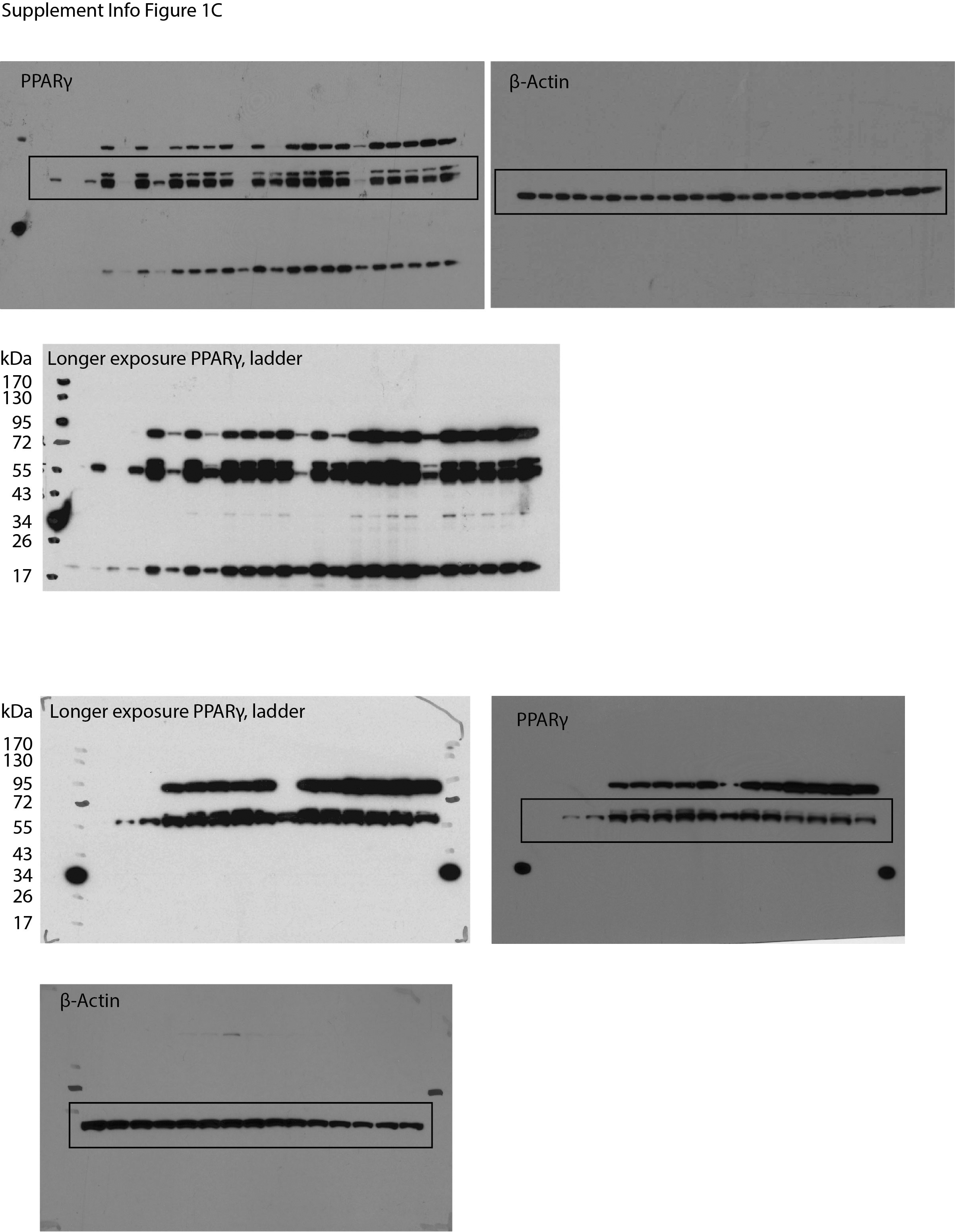


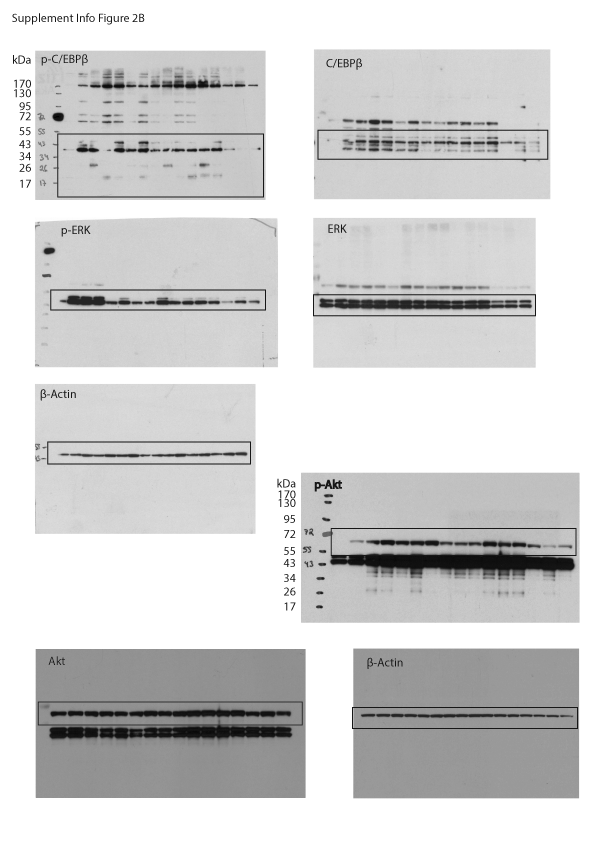


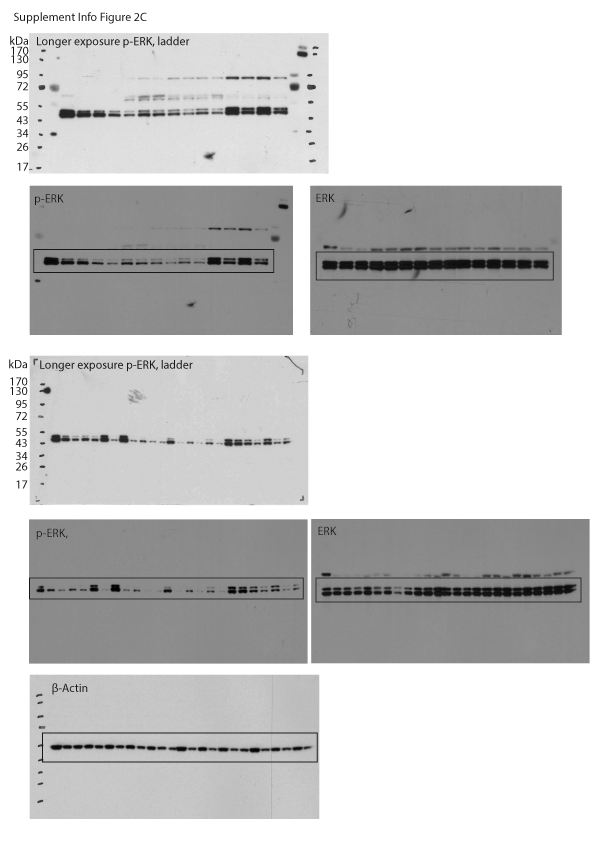


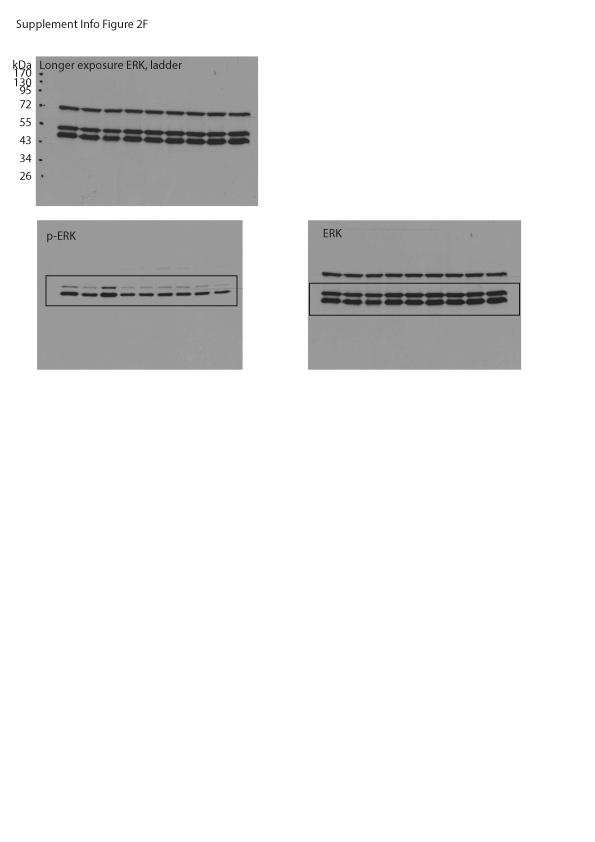


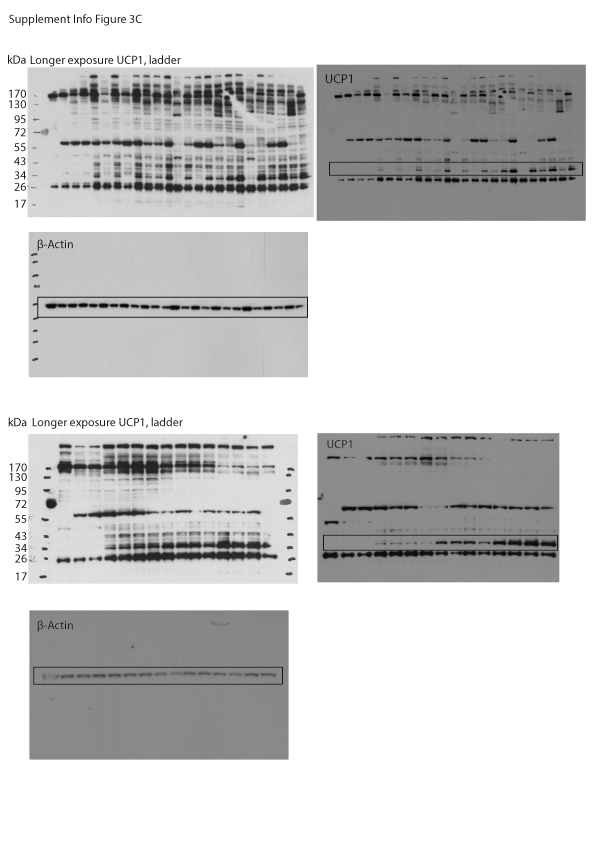


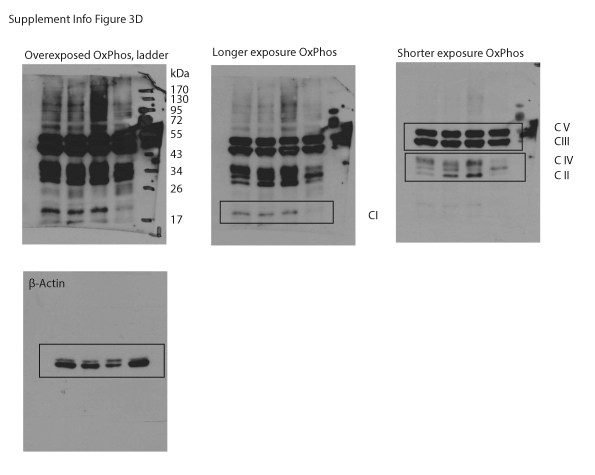


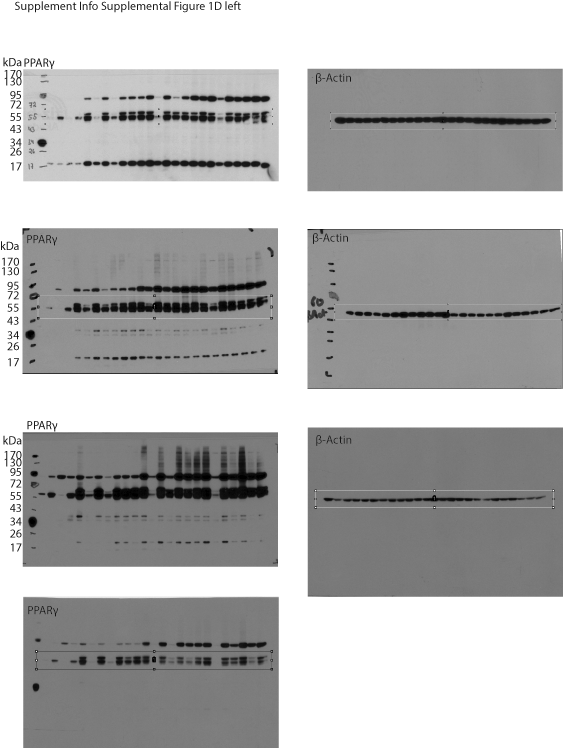


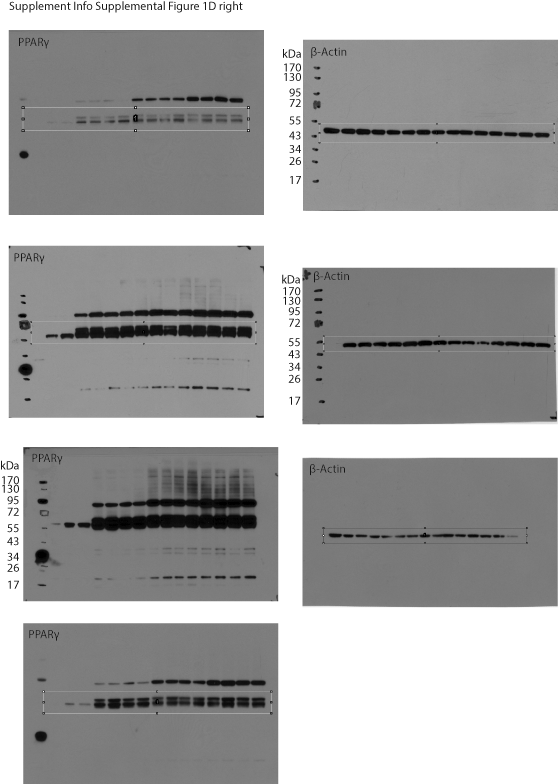


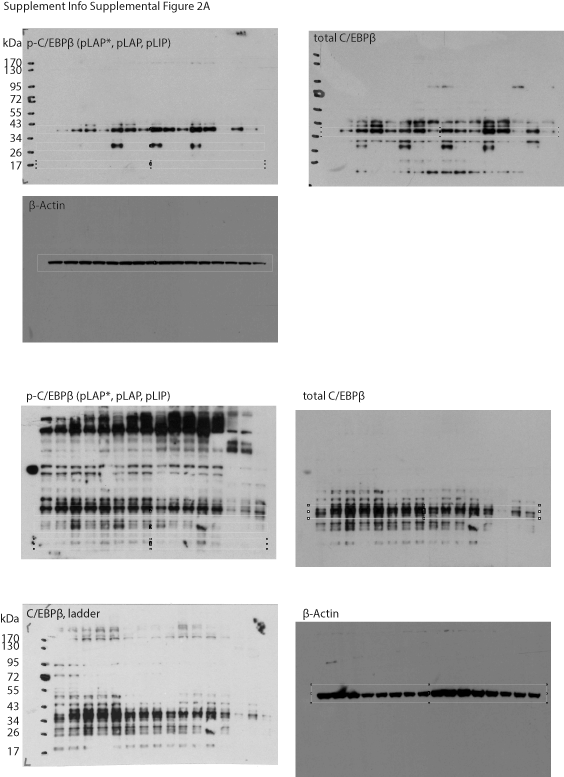


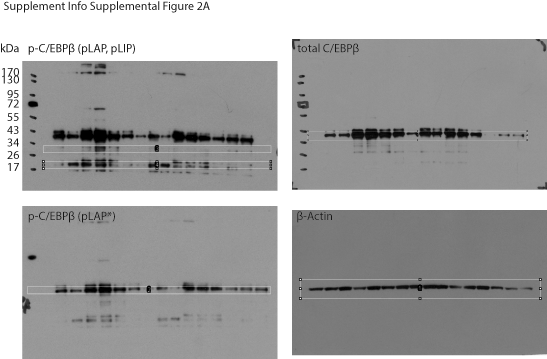


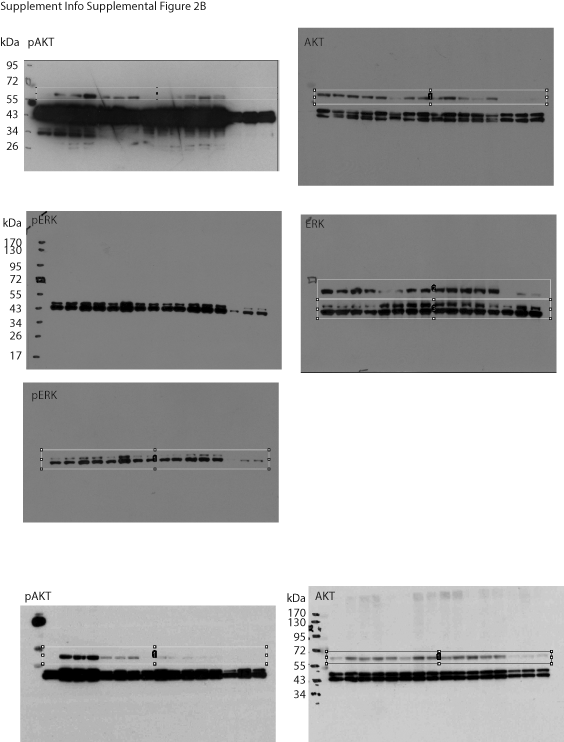


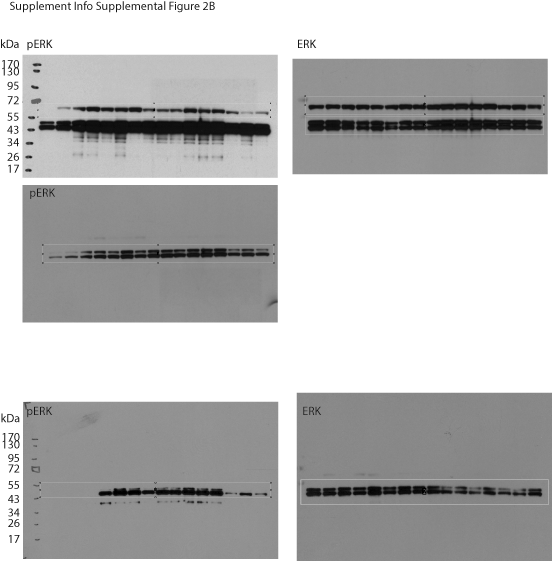


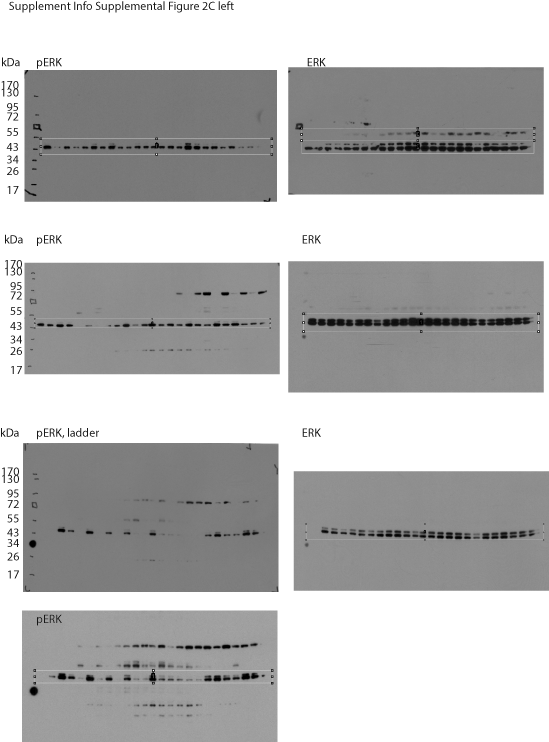


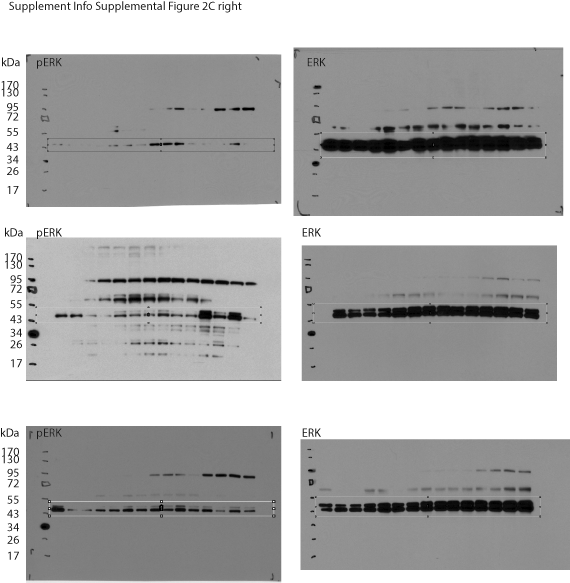


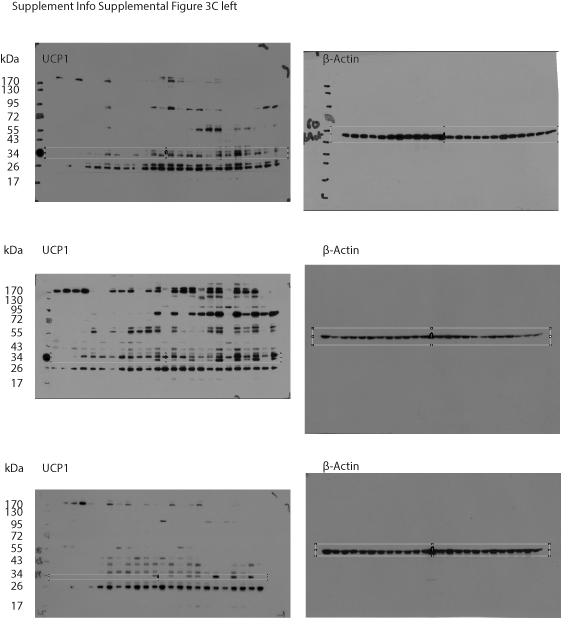


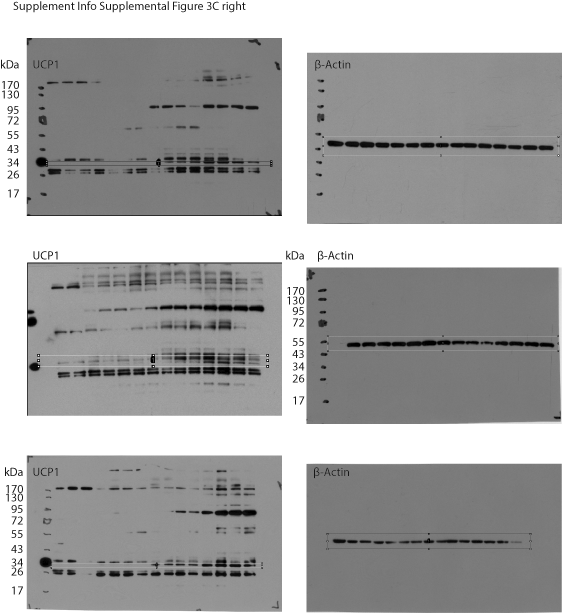


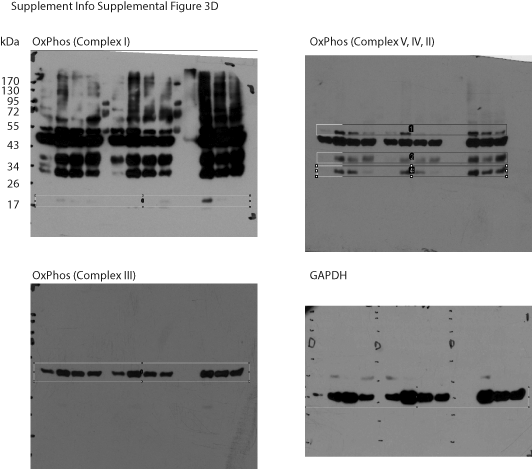

Supplement: Supplementary file 1 — Supplementary Information [file 41598_2017_9025_MOESM1_ESM.doc]
